# Guideline-concordant utilization of magnetic resonance imaging in adults receiving chiropractic manipulative therapy vs other care for radicular low back pain: a retrospective cohort study

**DOI:** 10.1186/s12891-022-05462-y

**Published:** 2022-06-08

**Authors:** Robert J. Trager, Brian R. Anderson, Regina M. Casselberry, Jaime A. Perez, Jeffery A. Dusek

**Affiliations:** 1 Connor Whole Health, University Hospitals Cleveland Medical Center, Cleveland, OH 44106 USA; 2 Palmer Center for Chiropractic Research, Palmer College of Chiropractic, Davenport, IA USA; 3 Clinical Research Center, University Hospitals Cleveland Medical Center, Cleveland, OH 44106 USA; 4 Department of Family Medicine and Community Health, School of Medicine, Case Western Reserve University, Cleveland, OH 44106 USA

**Keywords:** Services Utilization, Magnetic Resonance Images, Clinical Guideline, Chiropractic, Low back pain, Radiculopathy, Electronic Medical Records, Integrative Medicine

## Abstract

**Background:**

Lumbar magnetic resonance imaging (LMRI) is often performed early in the course of care, which can be discordant with guidelines for non-serious low back pain. Our primary hypothesis was that adults receiving chiropractic spinal manipulative therapy (CSMT) for incident radicular low back pain (rLBP) would have reduced odds of early LMRI over 6-weeks’ follow-up compared to those receiving other care (a range of medical care, excluding CSMT). As a secondary hypothesis, CSMT recipients were also expected to have reduced odds of LMRI over 6-months’ and 1-years’ follow-up.

**Methods:**

A national 84-million-patient health records database including large academic healthcare organizations (TriNetX) was queried for adults age 20–70 with rLBP newly-diagnosed between January 31, 2012 and January 31, 2022. Receipt or non-receipt of CSMT determined cohort allocation. Patients with prior lumbar imaging and serious pathology within 90 days of diagnosis were excluded. Propensity score matching controlled for variables associated with LMRI utilization (e.g., demographics). Odds ratios (ORs) of LMRI over 6-weeks’, 6-months’, and 1-years’ follow-up after rLBP diagnosis were calculated.

**Results:**

After matching, there were 12,353 patients per cohort (mean age 50 years, 56% female), with a small but statistically significant reduction in odds of early LMRI in the CSMT compared to other care cohort over 6-weeks’ follow-up (9%, 10%, OR [95% CI] 0.88 [0.81–0.96] *P* = 0.0046). There was a small but statistically significant increase in odds of LMRI among patients in the CSMT relative to the other care cohort over 6-months’ (12%, 11%, OR [95% CI] 1.10 [1.02–1.19], *P* < 0.0174) and 1-years’ follow-up (14%, 12%, OR [95% CI] 1.21 [1.13–1.31], *P* < 0.0001).

**Conclusions:**

These results suggest that patients receiving CSMT for newly-diagnosed rLBP are less likely to receive early LMRI than patients receiving other care. However, CSMT recipients have a small increase in odds of LMRI over the long-term. Both cohorts in this study had a relatively low rate of early LMRI, possibly because the data were derived from academic healthcare organizations. The relationship of these findings to other patient care outcomes and cost should be explored in a future randomized controlled trial.

**Registration:**

Open Science Framework (https://osf.io/t9myp).

**Supplementary Information:**

The online version contains supplementary material available at 10.1186/s12891-022-05462-y.

## Background

In the United States (US), chiropractors are often the first provider to see a patient with radicular low back pain (rLBP) [1], a form of low back pain (LBP) with nerve root involvement. As such, their initial management strategy may affect patients’ subsequent use of other health services such as imaging and prescriptions [2, 3]. In the integrative setting, chiropractors often function as spine care providers [4–7], and frequently receive referrals to evaluate and treat LBP [5]. While magnetic resonance imaging (MRI) of the lumbar spine (LMRI) is often overutilized for LBP, the association between chiropractic care and LMRI utilization is not well understood.

Although the rate of early advanced imaging (including LMRI) for LBP in primary care has increased in the 21st Century [8], such early imaging may have certain drawbacks among those with non-serious LBP. Early LMRI is associated with increased utilization of other diagnostic tests [9], injections [9], opioid prescriptions [10], and surgeries [9, 10], as well as increased work absences [9], length of work disability [11], and treatment costs [9, 10]. In addition, one study found that chiropractors’ referral for imaging (including MRI) at initial patient encounters was not associated with improved treatment outcomes [12].

According to imaging guidelines for LBP with or without radiculopathy, LMRI is considered appropriate when there is a clinical suspicion of serious pathology or presence of “red flag” signs or symptoms, such as trauma, malignancy, infection, or a focal neurologic deficit with progressive or disabling symptoms [13, 14]. Guidelines also regard persistent LBP (i.e., 4–8 weeks) and surgical candidacy as criteria for appropriate LMRI [14, 15]. Multiple professional US organizations, including the American Academy of Family Physicians [16] and The American Chiropractic Association [17], have issued statements that lumbar imaging (including LMRI) should be avoided during the first 6 weeks of care for LBP unless there are red flags or other specific indications for imaging.

Despite these recommendations, LMRI is often performed without guideline-concordant indications. Large studies within the US have estimated that LMRI for LBP is performed early or inappropriately in 22–35% of cases [18–21]. These findings are based on similar criteria to those used in the current study, such as symptom duration and red flags. Considering that chiropractors often treat patients with low back disorders, we sought to determine if LMRI utilization was likewise high in patients receiving this form of care.

In treatment of rLBP, chiropractors commonly administer spinal manipulative therapy (SMT) [22, 23], a type of manual therapy directed to the spinal joints [24]. SMT, also called chiropractic SMT (CSMT), is the most common treatment administered by chiropractors [25]. In addition, chiropractors are the predominant providers of SMT in integrative settings, and are often identified with this form of care [5].

In the US, chiropractors’ scope of practice includes the ability to order MRI [26]. Occasionally, this ability can be limited by individual insurers, such as Medicare [27]. Like other providers, chiropractors are encouraged to follow imaging guidelines when ordering LMRI [17]. Given chiropractors treat rLBP and in many cases may directly order LMRI, it is possible their management strategy would influence subsequent LMRI utilization.

Research regarding chiropractic care and MRI utilization is limited [3, 28, 29]. Two previous studies found that recipients of chiropractic care were less likely to undergo advanced imaging, however data for computed tomography (CT) and LMRI were combined, making it difficult to determine the independent likelihood of LMRI [3, 29]. While chiropractors have tended to overutilize radiography [30], this practice may not necessarily apply to LMRI, as a scoping review in 2017 found that chiropractors’ routine use of radiographs was much higher than that of MRI (i.e., 35% vs. 1%) [25]. The current study is unique in that it focuses on early LMRI utilization in a specific population of rLBP.

Considering the rate of LMRI has increased without corresponding improvement in patient outcomes, and chiropractors commonly manage LBP and may order LMRI, this study examined guideline-concordant utilization of LMRI among recipients of CSMT compared to recipients of other care (a range of medical care, excluding CSMT).

## Methods

### Objective

The objective of this study is to examine LMRI utilization among recipients of CSMT compared to those receiving other care, with the following hypotheses:

1. As a primary hypothesis, we expect adults receiving CSMT for incident rLBP will have a reduced odds of LMRI over 6-weeks’ follow-up from index diagnosis compared to those receiving other care.
2. As a secondary hypothesis, we expect CSMT recipients to have reduced odds of LMRI over 6-months’ and 1-years’ follow-up.

### Study design

**Fig. 1:**
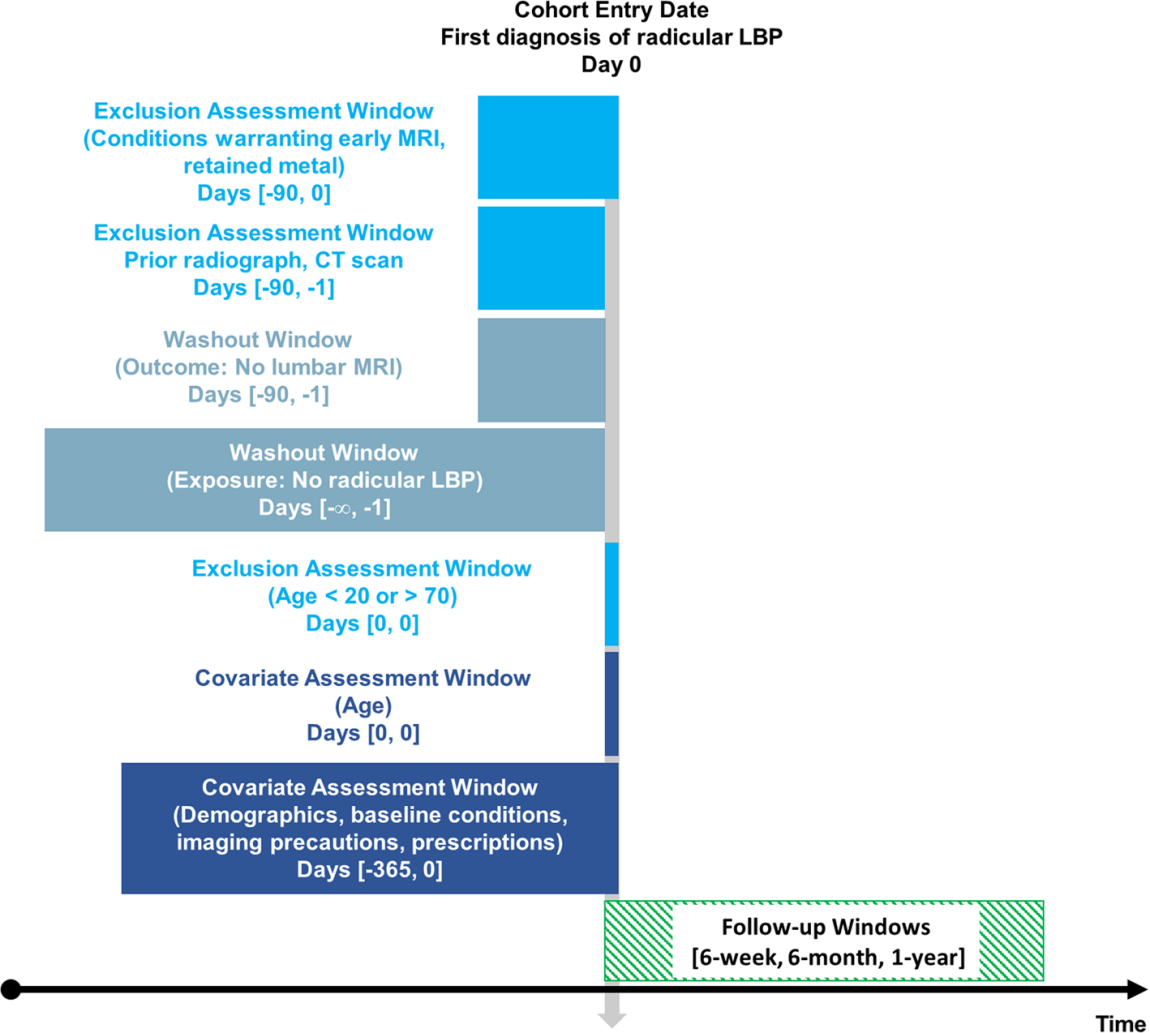
Graphical depiction of study design. The vertical grey arrow represents the index date when each individual patient was diagnosed with radicular low back pain (cohort entry date, day 0). Text to the left of this arrow describes study selection criteria which were assessed during time windows ([#, #]) of days preceding and the index date. Rectangles that overlap with the vertical grey line also overlap with the index diagnosis date (day 0). The washout period for radicular low back pain was infinite (∞). The Follow-up Windows are the only numbers not described in terms of days (6-weeks, 6-months, 1-year).. Abbreviations: computed tomography (CT), low back pain (LBP), magnetic resonance imaging (MRI), X-ray (XR). Image created using Creative Commons template from Schneeweiss et al. [34]

This study followed an a-priori protocol [31] which was modified to better assess for exclusions by including the date of index diagnosis rather than only up to the preceding day. The current study reporting adheres to the Strengthening the Reporting of Observational Studies in Epidemiology statement [32]. This study uses a retrospective cohort design (Fig. 1), incorporating real-world electronic health records data. A new-user design, including only new diagnoses of rLBP, was used to make cohorts more comparable and reduce bias [33]. The data query date was January 31, 2022, with a search window from January 31, 2012 to January 31, 2022. This date range was used to examine more recent data, considering imaging guidelines and related LMRI ordering practices may have changed over time. The current study was approved by the University Hospitals Institutional Review Board (STUDY20211554).

Although the current study methods of detecting early LMRI has similarities to measures from the National Quality Forum / Centers for Medicare & Medicaid Services [35] and National Committee for Quality Assurance [36], those measures were not ideally structured for our study as they focus on generalized, rather than radicular LBP. These measures are also intended for use with administrative claims rather health records data which were used in the current study. Although several methods exist for determining appropriateness of MRI, there is no clear consensus strategy [13, 37]. Customization of our methods of detecting LMRI utilization allowed for an approximation with several imaging guidelines, while using health records as a data source enabled several baseline differences between cohorts to be controlled for [38].

### Setting and data source

This study utilized a national research network (TriNetX, Inc., Cambridge, MA) which includes aggregated, de-identified health records data from multiple health care organizations (HCOs) [39]. At the time of query (January 31, 2022), the network included 84.2 million patients across 56 HCOs in the US, 10 of which included chiropractors. Included HCOs are large and academically affiliated yet remain anonymous for patient privacy. TriNetX allows queries using standardized terminologies such as the International Classification of Disease (ICD) codes. At University Hospitals of Cleveland, access to TriNetX is managed by the hospitals’ Clinical Research Center.

### Participants

#### Eligibility criteria

Eligibility criteria were designed to reflect previously published LMRI guidelines by excluding conditions warranting early appropriate LMRI within 6-weeks’ follow-up [13–15], and make cohorts more comparable by narrowing the study population to rLBP rather than all types of LBP.

This study included adults age 20–70, given that a younger or older age could warrant early appropriate LMRI [13]. Patients were included at their initial diagnosis date of rLBP such that they entered the follow-up window at a uniform time point. Radicular LBP was chosen as the target condition to standardize the study population based on LBP complexity. Including all types of LBP could introduce confounding by allowing for between-cohort differences with respect to LBP severity, which could in turn influence the likelihood of LMRI.

A phenotype, or definition, for rLBP was customized for this study as a previously validated phenotype specific to our study objectives did not exist. This was accomplished by adapting a generalized LBP phenotype to only include codes that described rLBP [40], as described in our protocol [31]. Diagnoses of spondylosis or disc degeneration were not included given these do not necessarily cause radicular pain [41]. Thoracic spine and sacroiliac joint diagnoses were not included given these were outside of the study focus.

Red-flag indicators of serious pathology that could warrant appropriate early (≤ 6 weeks) LMRI were excluded. Examples of such exclusions are [13]: abnormal weight loss, fever, malignancy, cauda equina syndrome, urinary/fecal incontinence, spinal fracture or infection, and trauma. In addition, patients with previous spinal surgery were excluded via diagnoses specifying postlaminectomy syndrome and arthrodesis status [13]. Patients with retained metal fragments were excluded as this is a contraindication to MRI.

Patients with prior lumbar radiography, CT, and LMRI over the 90-days preceding index diagnosis were excluded to further exclude those with pre-existing LBP (Fig. 1). These exclusions were applied so that patients in both cohorts would uniformly have no recent lumbar imaging at baseline. Lumbar imaging codes were used from a prior study [42]. This general strategy has been used in previous observational studies [3, 43].

#### Chiropractic care

Procedure codes specifying receipt of CSMT were used as a marker to identify recipients of chiropractic care. Patients were allocated into 2 cohorts according to receipt or non-receipt of CSMT, a strategy that has been used previously [44, 45]. These codes should be sufficiently specific to chiropractic care, as one study identified at least 97% of providers using these codes were chiropractors [46].

### Outcome variables

#### Lumbar magnetic resonance imaging

LMRI was identified using procedure codes from a prior study [42]. Code definitions were identified using a billing guidance website [47]. Multiple follow-up windows were used to identify LMRI. An early follow-up window of 6 weeks was used to examine guideline-concordance (e.g., appropriate vs. early), based on previous recommendations [16, 17, 48–50]. Long-term follow-up windows of 6 months and 1 year were used so results could be comparable to a previous study [3] as well as to examine potential changes in odds of LMRI over time.

The current study design did not require chiropractors to be the ordering provider of LMRI. This allowed flexibility in the case that chiropractors may refer patients to primary care or a specialist for LMRI ordering, or may be unable to order LMRI given insurance restrictions (e.g., Medicare) [27].

#### Confounding variables

Propensity score matching (PSM) was used to control for confounding variables present within 1 year preceding index rLBP diagnosis with the goal of making cohorts comparable [33] with regards to the likelihood of receiving LMRI. The literature was searched to identify variables associated with increased or decreased LMRI utilization. Identified variables were converted to standardized terminology for use in PSM via websites [51, 52].

Demographic variables including age, sex, race, and ethnicity were propensity matched given these may influence the odds of LMRI [53–55]. All diagnoses within the ICD-10 category for “Mental, Behavioral and Neurodevelopmental disorders” were propensity matched, given there is an association between mental health and early LMRI utilization [55].

Conditions that could prevent or delay LMRI ordering or scheduling were also propensity matched: claustrophobia, contrast allergy, and presence of a cardiac pacemaker or spinal cord stimulator. The presence of a neurostimulator or cardiac pacemaker was propensity matched as these devices can be a precaution to undergoing MRI [56].

Although intravenous drug abuse can warrant early appropriate LMRI, it has no specific available ICD-10 code to allow its exclusion [57]. As a workaround, matching for “Mental, Behavioral and Neurodevelopmental disorders” enabled a range of substance abuse disorders to be controlled for. Current use of corticosteroids was propensity matched, as an additional measure of control in the case that long-term corticosteroid recipients may not have been fully excluded by ICD-10 code. Opioid prescription was propensity matched given opioids have been associated with increased early LMRI utilization [10, 58].

### Study size

Our sample size calculation resulted in a required sample size of 1,756 based on LMRI utilization over 6 weeks, using data from a prior similar study of a broader LBP population [55]. Calculations were performed using G*Power (v 3.1.9.6, Universität Düsseldorf) z-tests for logistic regression with an α error of 0.05 and a power of 0.95, assuming a normal distribution. A value of 0.9 was used for R^2^, considering a potential high level of interaction between covariates, and odds ratio (OR) of 0.53 from the prior study [55]. The probability of LMRI given the alternative hypothesis was 0.198, the rate of LMRI utilization in LBP among all patients in the prior study [55].

### Statistical methods

All statistical analyses were conducted in real-time using TriNetX. Logistic regression was used to calculate propensity scores for each patient. Parameters of PSM included greedy nearest-neighbor matching with a 1:1 matching ratio and caliper of 0.01 pooled standard deviations. Baseline characteristics were compared using an independent-samples t-test for continuous variables and a Pearson χ^2^ test for categorical variables. Odds of LMRI per cohort were calculated by dividing the number of patients with LMRI by the number of patients without LMRI. Odds ratios for LMRI were calculated by dividing odds in the CSMT by other care cohort.

## Results

### Participants

A large population was available for each cohort (Table 1). Before PSM, there were 12,353 patients in the CSMT cohort and 1,145,802 in the other care cohort. After PSM, there were 12,353 per cohort (mean [SD] age, 50 years, 56% female). During matching, the larger other care cohort reduced in number to equal the CSMT cohort size as patients that did not match were discarded.

**Table 1:** Baseline cohort characteristics before and after propensity score matching

	Before Matching			After Matching		
Characteristic	CSMT	Other care	*P *- value	CSMT	Other care	*P* - value
N	12,353	1,145,802		12,353	12,353	
Age	50.4 ± 14.0	49.7 ± 13.1	< 0.001	50.4 ± 14.0	49.9 ± 14.0	0.002
Sex						
Female	6,922 (56%)	637,871 (56%)	0.417	6,922 (56%)	6,857 (56%)	0.405
Male	4,755 (38%)	474,502 (41%)	< 0.001	4,755 (38%)	4,783 (39%)	0.714
Unknown Sex	676 (5%)	33,429 (3%)	< 0.001	676 (5%)	713 (6%)	0.307
Race						
Black	491 (4%)	180,872 (16%)	< 0.001	491 (4%)	484 (4%)	0.819
White	8,890 (72%)	699,608 (61%)	< 0.001	8,890 (72%)	8,841 (72%)	0.489
Asian	93 (1%)	19,375 (2%)	< 0.001	93 (1%)	92 (1%)	0.941
Unknown Race	2,846 (23%)	240,834 (21%)	< 0.001	2,846 (23%)	2,889 (23%)	0.517
Ethnicity						
Hispanic/Latino	265 (2%)	84,587 (7%)	< 0.001	265 (2%)	270 (2%)	0.827
Not Hispanic/Latino	10,340 (84%)	766,429 (67%)	< 0.001	10,340 (84%)	10,333 (84%)	0.904
American Indian or Alaska Native	31 (< 1%)	3,929 (< 1%)	0.082	31 (< 1%)	45 (< 1%)	0.108
Native Hawaiian or Other Pacific Islander	10 (< 1%)	1,184 (< 1%)	0.441	10 (< 1%)	10 (< 1%)	1.000
Unknown Ethnicity	1,748 (14%)	294,786 (26%)	< 0.001	1,748 (14%)	1,750 (14%)	0.971
Conditions (ICD-10)						
Mental, Behavioral, and Neurodevelopmental Disorders (F01-F99)	4,118 (33%)	309,875 (27%)	< 0.001	4,118 (33%)	4,168 (34%)	0.500
Opioid Related Disorders (F11)	91 (1%)	11,833 (1%)	0.001	91 (1%)	91 (1%)	1.000
Claustrophobia (F40.240)	26 (< 1%)	1,500 (< 1%)	0.015	26 (< 1%)	24 (< 1%)	0.777
Radiographic Dye Allergy Status (Z91.041)	13 (< 1%)	1,610 (< 1%)	0.297	13 (< 1%)	25 (< 1%)	0.051
Presence of Cardiac Pacemaker (Z95.0)	11 (< 1%)	2,786 (< 1%)	0.001	11 (< 1%)	12 (< 1%)	0.835
Presence of Neurostimulator (Z96.82)	10 (< 1%)	114 (< 1%)	< 0.001	10 (< 1%)	10 (< 1%)	1.000
Medications (VANDF)						
Adrenal Corticosteroids (HS050)	4,441 (36%)	354,270 (31%)	< 0.001	4,441 (36%)	4,419 (36%)	0.770
Opioid Analgesics (CN101)	3,858 (31%)	351,548 (31%)	0.187	3,858 (31%)	3,872 (31%)	0.848

Abbreviations: Chiropractic spinal manipulative therapy *CSMT*, International Classification of Diseases *ICD*, Veterans Health Administration National Drug File *VANDF*

Before PSM, the CSMT cohort had a greater proportion of patients who were White, and not Hispanic/Latino compared to the cohort receiving other care. Additionally, the CSMT cohort had a greater frequency of diagnoses within the ICD-10 category “Mental, Behavioral, and Neurodevelopmental Disorders,” as well as use of adrenal corticosteroids. After PSM, these variables were not significantly different between cohorts (*P* > 0.05).

### Descriptive data

The number of data points per patient in each cohort was high (CSMT 2,880; other care 1,343) suggesting a low likelihood of bias related to missing information. A visual diagnostic showed that cohort propensity scores were adequately matched.

### Key results

After PSM, patients in the CSMT cohort had a small [59] but significantly reduced odds of early LMRI compared to the other care cohort through 6-weeks’ follow-up (Table 2; 9%, 10%, OR [95% CI] 0.88 [0.81–0.96] *P* = 0.0046). There was a small but significant increase in odds of LMRI among patients in the CSMT relative to the other care cohort over 6-months’ (12%, 11%, OR [95% CI] 1.10 [1.02–1.19], *P* < 0.0174) and 1-years’ follow-up (14%, 12%, OR [95% CI] 1.21 [1.13–1.31], *P* < 0.0001).

**Table 2:**
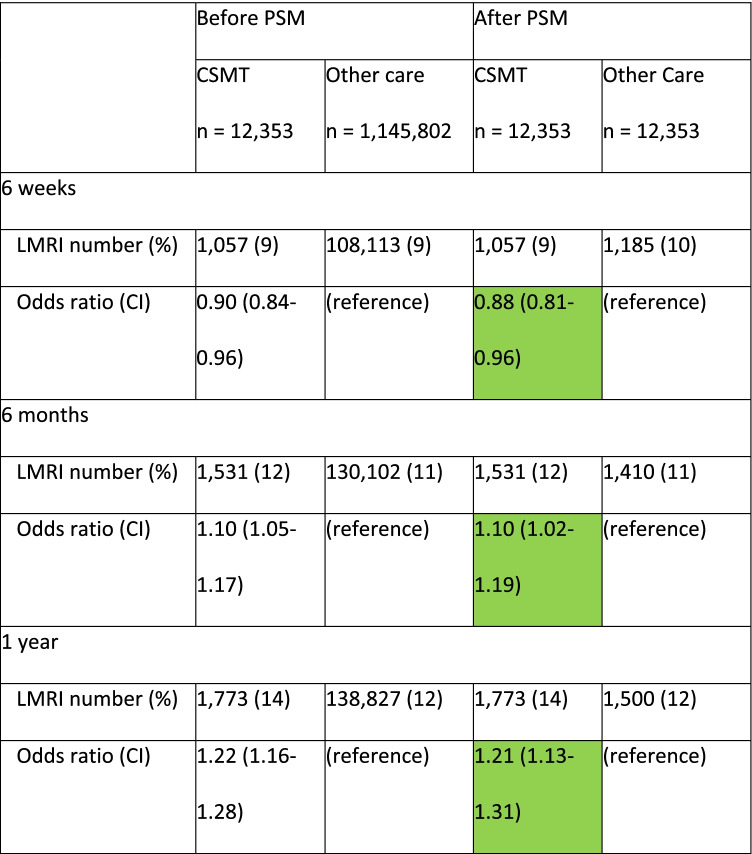
Key results

Cells with a green background contain odds ratios and their confidence intervals pertinent to the study hypotheses

Abbreviations: 95% confidence intervals *CI*, chiropractic spinal manipulative therapy *CSMT*, odds ratio *OR*, propensity score matching *PSM*, and percentage (%) of patients with lumbar magnetic resonance imaging *LMRI*

## Discussion

This study examined LMRI utilization among patients with rLBP initially receiving either CSMT or other care, using a large, propensity-matched sample. Multiple follow-up windows were used, including a window of 6-weeks, reflective of LMRI appropriateness and imaging guideline-concordance, and longer-term follow-ups of 6-months and 1-year. Study strengths included the use of multicenter, national data, with extensive selection criteria and propensity matching that controlled for several confounding variables.

As recipients of CSMT had reduced odds of early LMRI compared those receiving other care, the primary study hypothesis is accepted. However, the secondary hypothesis is rejected as those receiving CSMT had an increased odds of LMRI utilization over 6-months’ and 1-years’ follow-up. The magnitude of differences in the percentage of LMRI utilization between cohorts in the current study were small over each follow-up window (i.e., 1–2%), yet statistical significance was reached due to the large sample size. Accordingly, further research is needed to determine if these small differences would translate to clinically meaningful changes in patient treatment outcomes or cost of care. Specifically, a randomized controlled trial could be used to examine the likelihood of LMRI in parallel with cost of treatment and changes in patient disability among those receiving CSMT versus other care.

The current study results suggest that overutilization of LMRI was uncommon in the studied healthcare setting. The rate of early (≤ 6-weeks’) LMRI in this study was low for both cohorts (≤ 10%) compared to previous estimates using large, US-based samples (22–35%) [18–21]. In addition, rates of LMRI for both cohorts through 1-years’ follow-up in the current study (~ 12–14%) also remained lower than these previous estimates.

While the low rates of LMRI in this study could result from differences in study design (e.g., selection criteria), this could also be explained by the study setting involving large, academically-affiliated HCOs. One study found that providers in a group of more than 5 physicians ordered significantly less CT and MRI for acute LBP compared to solo practitioners [54]. Another qualitative study suggested that providers within academic settings could have greater LMRI guideline-concordance due to an increased ability to spend time with patients and conduct in-depth examinations [60].

Other organizational or financial explanations may explain the low rates of LMRI in the current study. Large academic HCOs such as those included in the current study may participate in Accountable Care Organizations within the Medicare Shared Savings Program [61], which offers financial incentives for system-wide imaging guideline-concordance [62]. HCO participation in this program has been associated with reduced MRI utilization [61, 63]. Further, clinician decision-support tools [21, 64] or LBP treatment guidelines internal to the HCO may have been available as a means to foster guideline-concordant imaging utilization.

In the current study, CSMT recipients had a small [59] increase in odds of LMRI compared to those receiving other care over 6-months’ to 1-years’ follow-up. This is not reflective of non-concordance with imaging guidelines, which consider persistent symptoms of this duration an acceptable indication for LMRI [14, 15]. Further, the higher percentage of LMRI in those receiving CSMT in the current study may not directly reflect increased ordering by chiropractors. Instead, patients receiving CSMT may have received more referrals for LMRI over the long term, as ordered by any treating provider, compared to those that received other care.

While prior studies found that patients receiving chiropractic care for LBP had reduced advanced imaging utilization over long-term follow-up [3, 29], the current study found a small [59] increase in odds of LMRI among patients with rLBP over 6-months to 1-year. The difference between the current results and those of prior studies could be explained by aspects of study design. For example, the current study focuses on rLBP rather than all types of LBP, and is limited to LMRI rather than multiple forms of advanced imaging (CT or MRI).

### Limitations

First, as an observational study, it is not possible to determine that CSMT or other care was responsible for causing differences in LMRI utilization. Unmeasured confounders may have affected results, chiefly socioeconomic variables, which are not well-represented in the TriNetX database.

Second, patients could have been misclassified who had chronic rLBP and/or prior LMRI. This could occur if patients presented for care after being treated at an HCO outside of the TriNetX network. Additionally, red flags of serious pathology may not have been fully excluded, as these may have been not documented in the medical record. We were unable to validate the accuracy of the current study rLBP phenotype against a gold-standard of chart review given it involved de-identified data from outside HCOs.

Third, due to limitations in data granularity, we were unable to propensity match for pain severity, neurological signs and symptoms, and LBP-related disability using a standardized score, each of which could influence likelihood of LMRI [53, 55]. We were also unable to compare LMRI utilization among chiropractors to other specific provider types (e.g., primary care, surgeons, other specialists) as provider codes are unavailable in TriNetX. Prior research has shown that LMRI utilization varies between provider types [21, 53].

Fourth, the study design (e.g., PSM, time windows, selection criteria) was tailored to LMRI utilization which precluded the ability to examine other outcomes such as radiograph or CT utilization, cost of LBP-related care, or surgery. A randomized controlled trial would enable better control of confounders and enable several such outcomes beyond LMRI to be measured in parallel.

Fifth, a small percentage of chiropractic patients not receiving CSMT may have been omitted. Omission of these patients would be unlikely to affect study results as contraindications to CSMT, such as spinal metastasis, infection, and other serious pathology [65] were also study exclusions.

Sixth, these results apply to chiropractors practicing within large, academically-affiliated HCOs and may not be generalizable to those in private practice. Only a minority (5.4%) of chiropractors in the US practice in integrative HCOs such as those in the TriNetX network [1]. Accordingly, this study may require replication in other healthcare settings and also within a randomized controlled trial.

## Conclusions

Patients initially receiving CSMT for rLBP had a small reduction in odds of early LMRI compared to those receiving other care, which was suggestive of imaging guideline concordance. Over long-term follow-up, CSMT recipients had a small increase in odds of LMRI. Overall, the rate of early LMRI was low in both cohorts, which could be explained by data being derived from large, academically affiliated HCOs. It is unclear if the small associations in this study correspond to meaningful differences in other patient outcomes. These findings should be explored in a future randomized controlled trial examining LMRI utilization alongside additional endpoints such as patient care outcomes and cost.

## Supplementary Information

**Additional file 1: Table 1.** Radicular low back pain inclusion codes with Boolean “OR”. **Table 2.** Exclusions for both cohorts, and corresponding assessment window relative to index diagnosis of radicular low back pain. **Table 3.** Additional inclusion/exclusion codes based on receipt of spinal manipulative therapy. **Table 4.** Magnetic resonance imaging codes. **Table 5.** Variables to be controlled for in propensity score matching. **Figure 1.** Cohort propensity score before matching (left) and after matching (right). Purple is the chiropractic cohort; green is the other care cohort. The propensity scores are similar before matching and overlap even more closely after matching, with no observable difference between cohorts.

## Data Availability

Study data obtained via the TriNetX network is subject to a data use agreement that does not permit it to be shared or released. Individuals may contact TriNetX to inquire about network access (https://www.trinetx.com/).

## Abbreviations

CSMT: Chiropractic spinal manipulative therapy
CI: Confidence interval
CT: Computed tomography
HCO: Healthcare organization
ICD: International Classification of Disease
LBP: Low back pain
LMRI: Lumbar magnetic resonance imaging
MRI: Magnetic resonance imaging
OR: Odds ratio
rLBP: Radicular low back pain
SD: Standard deviation
SMT: Spinal manipulative therapy
XR: X-ray
