# Supplementary material for: Guideline-concordant utilization of magnetic resonance imaging in adults receiving chiropractic manipulative therapy vs other care for radicular low back pain: a retrospective cohort study
Source: BMC Musculoskelet Disord. 2022 Jun 8;23:554. doi: 10.1186/s12891-022-05462-y (PMC9175310; doi:10.1186/s12891-022-05462-y)
Supplement: Supplementary file 1 — Additional file 1: Table 1. Radicular low back pain inclusion codes with Boolean “OR”. Table 2. Exclusions for both cohorts, and corresponding assessment window relative to index diagnosis of radicular low back pain. Table 3. Additional inclusion/exclusion codes based on receipt of spinal manipulative therapy. Table 4. Magnetic resonance imaging codes. Table 5. Variables to be controlled for in propensity score matching. Figure 1. Cohort propensity score before matching (left) and after matching (right). Purple is the chiropractic cohort; green is the other care cohort. The propensity scores are similar before matching and overlap even more closely after matching, with no observable difference between cohorts. [file 12891_2022_5462_MOESM1_ESM.docx]

# Additional file 1

Table 1: Radicular low back pain inclusion codes with Boolean “OR”

| **ICD-10 Code** | **ICD-10 Definition** |
| --- | --- |
| G54.4 | Lumbosacral root disorders, not elsewhere classified |
| M54.16 | Radiculopathy, lumbar region |
| M54.17 | Radiculopathy, lumbosacral region |
| M54.18 | Radiculopathy, sacral and sacrococcygeal region |
| M48.062 | Spinal stenosis, lumbar region with neurogenic claudication |
| M54.4 | Lumbago with sciatica |
| M54.3 | Sciatica |
| Abbreviations: International classification of diseases (ICD) | |

Table 2: Exclusions for both cohorts, and corresponding assessment window relative to index diagnosis of radicular low back pain

| **ICD-10 Code** | **ICD-10 Definition** |
| --- | --- |
| Conditions warranting appropriate early lumbar MRI, excluded days -90 to 0 | |
| B20 | Human immunodeficiency virus [HIV] disease |
| C00-C96 | Malignant neoplasm |
| D84 | Other immunodeficiencies |
| G06 | Intracranial and intraspinal abscess and granuloma |
| G83.4 | Cauda equina syndrome |
| M05 | Rheumatoid arthritis with rheumatoid factor |
| M06 | Other rheumatoid arthritis |
| M45 | Ankylosing spondylitis |
| M46 | Other inflammatory spondyloarthropathies (includes infections) |
| M48.4 | Fatigue fracture of vertebra |
| M48.5 | Collapsed vertebra, not elsewhere classified |
| M80 | Osteoporosis with current pathological fracture |
| M81 | Osteoporosis without current pathological fracture |
| M96.1 | Postlaminectomy syndrome, not elsewhere classified |
| R15 | Fecal incontinence |
| R32 | Unspecified urinary incontinence |
| R50 | Fever of other and unknown origin |
| R63.4 | Abnormal weight loss |
| S30-S39 | Injuries to the abdomen, lower back, lumbar spine, pelvis and external genitals |
| Z79.52 | Long term (current) use of systemic steroids |
| Z98.1 | Arthrodesis status |
| Contraindications to imaging, excluded days -90 to 0 | |
| Z18.1 | Retained metal fragments |
| Prior lumbar spine radiograph and computed tomography (CPT codes), excluded days -90 to -1 | |
| 72081-4 | Radiologic examination, spine, entire thoracic and lumbar, including skull, cervical, and sacral spine if performed |
| 72020 | Radiologic examination, spine, single view, specify level |
| 72100 | Radiologic examination, spine, lumbosacral, 2 or 3 views |
| 72110 | Radiologic examination, spine, lumbosacral, minimum of four views |
| 72114 | Radiologic examination, spine, lumbosacral; complete, including bending views, minimum of 6 views |
| 72120 | Radiologic examination, spine, lumbosacral, bending views only, 2 or 3 views |
| 72200 | Radiologic examination, sacroiliac joints; less than 3 views |
| 72202 | Radiologic examination, sacroiliac joints; 3 or more views |
| 72131 | Computed tomography, lumbar spine; without contrast material |
| 72132 | Computed tomography, lumbar spine; with contrast material |
| 72133 | Computed tomography, lumbar spine; without contrast material, followed by contrast material(s) and further sections |
| Prior lumbar spine MRI (CPT codes), excluded days -90 to -1 | |
| 72148 | Magnetic resonance (eg, proton) imaging, spinal canal and contents, lumbar; without contrast material |
| 72149 | Magnetic resonance (eg, proton) imaging, spinal canal and contents, lumbar; with contrast material(s) |
| 72158 | Magnetic resonance (eg, proton) imaging, spinal canal and contents, without contrast material, followed by contrast material(s) and further sequences; lumbar |
| Healthcare services (CPT), excluded days -90 to 0 | |
| 99291-99292 | Critical care |
| 99221-99239 | Hospital inpatient services |
| Abbreviations: Current procedural terminology (CPT), human immunodeficiency virus (HIV), International classification of diseases (ICD), magnetic resonance imaging (MRI). | |

Table 3: Additional inclusion/exclusion codes based on receipt of spinal manipulative therapy

| **CPT code** | **Definition** | **Recipients of CSMT** | **Nonrecipients of CSMT** |
| --- | --- | --- | --- |
| 98940 | CSMT; spinal, 1-2 regions | Included | Excluded |
| 98941 | CSMT; spinal, 3-4 regions | Included | Excluded |
| 98942 | CSMT; spinal, 5 regions | Included | Excluded |
| Abbreviations: Chiropractic spinal manipulative therapy (CSMT) | | | |

Table 4: Magnetic resonance imaging codes

| **Code** | **Description** |
| --- | --- |
| 72148 | Magnetic resonance (eg, proton) imaging, spinal canal and contents, lumbar; without contrast material |
| 72149 | Magnetic resonance (eg, proton) imaging, spinal canal and contents, lumbar; with contrast material(s) |
| 72158 | Magnetic resonance (eg, proton) imaging, spinal canal and contents, without contrast material, followed by contrast material(s) and further sequences; lumbar |

Table 5: Variables to be controlled for in propensity score matching

| **Variable** | **Description** |
| --- | --- |
| Demographics | Patient age, sex, race, and ethnicity |
| Diagnoses (ICD-10) | |
| F01-F99 | Mental, Behavioral and Neurodevelopmental disorders |
| F11 | Opioid related disorders |
| Imaging precautions | |
| F40.240 | Claustrophobia |
| Z91.041 | Radiographic dye allergy status |
| Z95.0 | Presence of cardiac pacemaker |
| Z96.82 | Presence of neurostimulator |
| Prescribed medications (VANDF Classes) | |
| CN101 | Opioid analgesics |
| HS050 | Adrenal corticosteroids |
| Abbreviations: Central nervous system (CNS); Veterans Health Administration National Drug File (VANDF) | |


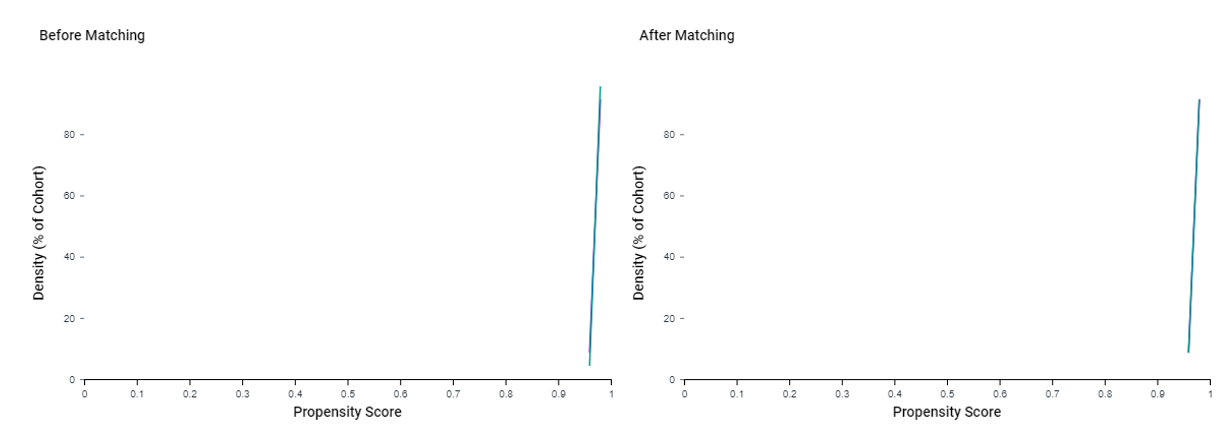


Figure 1: Cohort propensity score before matching (left) and after matching (right). Purple is the chiropractic cohort; green is the other care cohort. The propensity scores are similar before matching and overlap even more closely after matching, with no observable difference between cohorts.
